# Notice to Dyspeptics

**Published:** 1876-10

**Authors:** 


### *DYSPEPTICS MARK THIS!*

There is nothing tones the system like iron; there is nothing purifies the blood like sulphur. In every healthy person's organization iron is incorporated. Deprived of this metallic constituent the digestive apparatus and the secretive organs can not vigorously perform their functions. Supply the loss artificially by taking STAFFORD'S IRON AND SULPHUR POWDERS. The sulphur will purge the vitiative blood of impurities, the iron will invigorate the blood producing organs. If the complexion is muddy or sallow it will be rendered fresh and transparent. These results are guaranteed.

Sold by Druggists. 1 Package, 12 Powders, $1; 6 Packages, 72 Powders, $5. Mailed Free. HALL & RUCKEL, 218 Greenwich Street, N. Y.

